# Orthology prediction at scalable resolution by phylogenetic tree analysis

**DOI:** 10.1186/1471-2105-8-83

**Published:** 2007-03-08

**Authors:** René TJM van der Heijden, Berend Snel, Vera van Noort, Martijn A Huynen

**Affiliations:** 1 Center for Molecular and Biomolecular Informatics, Nijmegen Center for Molecular Life Sciences, Radboud University Nijmegen Medical Center, Nijmegen, The Netherlands

## Abstract

**Background:**

Orthology is one of the cornerstones of gene function prediction. Dividing the phylogenetic relations between genes into either orthologs or paralogs is however an oversimplification. Already in two-species gene-phylogenies, the complicated, non-transitive nature of phylogenetic relations results in inparalogs and outparalogs. For situations with more than two species we lack semantics to specifically describe the phylogenetic relations, let alone to exploit them. Published procedures to extract orthologous groups from phylogenetic trees do not allow identification of orthology at various levels of resolution, nor do they document the relations *between *the orthologous groups.

**Results:**

We introduce "*levels of orthology*" to describe the multi-level nature of gene relations. This is implemented in a program LOFT (Levels of Orthology From Trees) that assigns hierarchical orthology numbers to genes based on a phylogenetic tree. To decide upon speciation and gene duplication events in a tree LOFT can be instructed either to perform classical species-tree reconciliation or to use the species overlap between partitions in the tree. The hierarchical orthology numbers assigned by LOFT effectively summarize the phylogenetic relations between genes. The resulting high-resolution orthologous groups are depicted in colour, facilitating visual inspection of (large) trees. A benchmark for orthology prediction, that takes into account the varying levels of orthology between genes, shows that the phylogeny-based high-resolution orthology assignments made by LOFT are reliable.

**Conclusion:**

The "*levels of orthology*" concept offers high resolution, reliable orthology, while preserving the relations between orthologous groups. A Windows as well as a preliminary Java version of LOFT is available from the LOFT website .

## Background

Gene function prediction relies heavily on proper orthology prediction [1]. High quality orthology is not only essential for reliable annotation transfer, but also for predicting protein function by the co-occurrence of genes [2], predicting the effect of mutations [3], or the detection of subtle functional signals in the DNA [4]. Crudely speaking, there are two approaches for orthology prediction: best hit-based clustering methods, and tree-based methods. Best hit-based methods cluster the most similar genes in orthologous groups. Best hit-based methods are generally fast. They differ in their specific clustering rules but may allow the addition of genomes after orthologous groups have been established, without a complete reprocessing of the sequences. Examples of group orthology are COG [5] and KOG [6] and Markov Chain Clustering [7]. These methods tend to result in rather inclusive groups that may hold many paralogous genes within the same cluster. One specific cause of too inclusive orthologous groups in best-hit methods is gene loss of outparalogs in two species, causing the remaining outparalogs to become best bidirectional hits. Dessimoz et al. [8] have introduced a method to address this issue that uses the relative levels of sequence identity to so-called *witness *genes from a third species to detect cases of wrongly assigned orthology. Another best-hit based method, InParanoid [8,9], is much less inclusive as it is only defined for pair-wise comparison of genomes. In general, gene duplication followed by differential gene loss and/or varying rates of evolution can easily lead to wrong, or inclusive orthologous groups in best-hit based methods.

Tree-based methods [10-13] suffer less from differential gene-loss and varying rates of evolution than best-hit methods and offer, in principle, the highest resolution of orthology. In tree-based methods, one first has to establish the root of the tree. This is preferably done by using a known outgroup. Yet, outgroups must be selected carefully [14-16], making the criterion less useful in automated large scale analysis. The outgroup species may e.g. not be present in some of the gene-families, or, when using several outgroup species, their genes may not always cluster together. In addition, when analyzing species that cover all kingdoms, an outgroup species does not exist [16]. In those cases, one can e.g. use the longest branch as the root [17,18], midpoint rooting, gene tree parsimony [19], or a combination of methods. After deciding on the root of the tree, for each node must be established whether it represents a speciation event or a duplication event. To discriminate speciation from duplication events, species phylogenies can be mapped onto phylogenetic gene trees. Several automatic tree analysis methods have been described [13,17,19-23]. Mismatches between the trusted species tree and sections of the gene tree are interpreted as duplication events followed by gene losses. Optionally, one can require that mismatches are supported by bootstrapping techniques [13,23].

Instead of performing trusted species tree reconciliation, one can also use a simple "species-overlap" rule to decide whether nodes represent gene duplication or speciation events: a node is considered to represent a speciation event if its branches have mutually exclusive sets of species. Using an orthology benchmark, we will show that this species overlap rule performs remarkably well, especially considering its simplicity.

**Figure 1:**
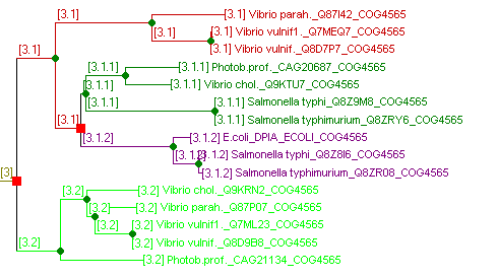
**Levels Of Orthology From Trees**. Genes in a subsection of the tree for COG4565 (transcription regulatory protein Dpia) have been numbered according their levels of relatedness. The tree has been analyzed for gene duplication (red squares) and speciation events (green circles), after which the numbering scheme ([3.1], [3.1.1] etc.) of LOFT gives a formal description of the levels of relatedness between the genes that allows a multi-level, scalable orthology. Only the part of the tree from COG4565, containing one of its orthologous groups ([3]) is shown.

Irrespective of how is decided whether nodes in a tree represent speciation or gene duplication events, the phylogenetic relations between genes can be pretty complicated. The terms ortholog, paralog and even inparalog, outparalog, and co-ortholog [1,8,24-26], defined to describe gene relations in pair-wise genome comparisons, are hardly sufficient to adequately describe them in case of multiple species comparisons. As an example of this is shown in Figure 1, a section of the tree for COG4565 that contains the genes from orthologous group 3 from this COG. Genes in orthologous group 3.1 are paralogous to genes in group 3.2, and genes in paralogous groups 3.1.1 and 3.1.2 are both orthologous to genes in group 3.1. Genes in groups 3.1.1 and 3.1.2 are outparalogous to each other because the duplication that separates these groups precedes the speciation events. Not only are genes from group 3.1.2 outparalogs to 3.1.1, but also genes from group 3.2. It is hard to specify in words that 3.1.2 is closer related to 3.1.1 then is 3.2. Deeper nesting makes these relations even more difficult to describe, as paralogous genes may split off at different levels and one ends up with different degrees of in- and outparalogy. This discussion demonstrates that an accurate verbose description of gene relations can be quite difficult and confusing. This has been recognized by others [13,25-27], but a solution has not yet been provided.

To describe and understand complicated phylogenetic situations as the one above, one generally has to resort to drawing the phylogenetic tree. However for describing phylogenetic relations and for automatic, large-scale analysis, the tree may not be an appropriate format. We therefore introduce the *levels of orthology *concept: a numbering scheme for describing relations between genes that can e.g. be used for automated phylogenomics. These LOFT numbers also capture the non-transitive nature of orthology (Figure 1): although genes from groups 3.1.1 and 3.1.2 are both orthologous to existent genes in group 3.1, they are paralogous to each other.

Note that in Figure 1 the genes *Photob.prof._CAG20687 *and *Vibrio chol._Q9KTU7 *in cluster 3.1.1 could easily have been misplaced. Given that the 3.1.1/3.1.2 split relies on a very short branch (the 3.1.1 root), they might well belong to cluster 3.1.2 instead. Such errors in tree topology may result in erroneous orthology assignments, underscoring the sensitivity of tree-based orthology to errors in the tree. However, by maintaining the relative relations between orthologous groups in the LOFT numbers, that in this case indicates a close relation between orthologous groups 3.1.1 and 3.2.1, the situation is less troublesome than if these relations would not have been maintained and all, and 3.1.1 would have been considered as different from 3.1.2 as from 3.2.

The "*levels of orthology*" concept, in combination with the simple species overlap rule, is implemented in a software tool, LOFT (Levels of Orthology From Trees). LOFT colors the various orthologous groups in a phylogenetic tree, strongly facilitating their recognition, especially in large trees. Some additional features improve the practicality of the tool, e.g. the option to highlight a certain gene or group of genes which helps to rapidly localize them in large trees. To assess the value of these high-resolution multi-level orthology assignments, we develop a benchmark for orthology prediction based on gene-order conservation. This benchmark is also sensitive to errors in reconstructed tree topologies that result in erroneous placement of duplication events. The results show a high correlation between phylogeny based orthology as implemented in LOFT and gene-order conservation.

## Results

LOFT was made to facilitate the analysis of phylogenetic trees. By its basic phylogenetic analysis, annotation of duplication and speciation nodes, and assignment of LOFT numbers, in combination with the functional use of color, it can be very helpful, especially when dealing with large trees. The hierarchical numbering scheme, that preserves the relations between orthologous groups, is comparable to the classification system in E.C. numbers or G.O. clusters. It therefore not only provides a scalable resolution to orthology, but also allows exporting the orthologous relations in a simple but powerful format, which is suited for large scale automated analysis.

### Levels of Orthology

Orthologous genes are only separated by speciation events. They include ancestral genes, and thus intermediate branches in the tree. By this simple observation, the concept of "*levels of orthology*" naturally arises. If an ancestral gene is assigned an orthology number, all genes, extinct or existent, that are separated from this gene by speciation events must be members of the same orthologous group. Accordingly, they must get the same orthology number. In contrast, a duplication event results in paralogous genes. A duplication event within a lineage causes an orthologous group to be split into two groups, which we call here '*sub-orthologous groups*'. The initial gene duplicates (the paralogs) can be seen as the first members of these sub-orthologous groups that form a new level. The example in Figure 1, showing a section of the tree for COG4565, shows two genes that originate through a duplication event from a gene in orthologous group 3. These paralogous lineages therefore receive numbers 3.1 and 3.2 respectively. Orthologous groups 3.1.1 and 3.1.2 are sub-orthologs from 3.1. Note that genes with orthologous level 3.1 may exist in parallel to genes in groups 3.1.1 and 3.1.2. This occurs in lineages where no further gene duplications have occurred (lineage to existent genes 3.1), while in others there have (the lineages to genes 3.1.1 and 3.1.2). Groups like group 3, which derive directly from ancient duplications, are referred to as base groups.

All complicated evolutionary relations between genes can be elegantly described using this concept of orthology levels. Genes that have the same orthology number (e.g. 3.1) are full and ordinary orthologs. Genes with numbers 3.1.1 and 3.1.2 have a paralogous relation with each other, as both descend from a duplication of an ancestral gene with orthology number 3.1. Accordingly, these genes are orthologous to genes with orthology number 3.1, their direct shared parent-group. The paralogous relation between genes with numbers 3.1.1 and 3.1.2 also holds for genes in different species. To our opinion, the level of orthology concept is very informative as it offers both a high resolution orthology and a discretization of the level of relatedness between different orthologous groups.

### Benchmarking Orthology Assignment

To assess the quality of these LOFT assignments, we developed a benchmark. In contrast to homology, where 3D structure can be used to assess the quality of predictions, there is no independent information to decide whether genes are orthologous to each other. We therefore developed an orthology benchmark based on internal consistency: to what extent do we observe gene-order conservation between groups of orthologous genes?

The benchmark examines the internal consistency between assigned orthology and gene neighborhood. Gene-order conservation is considered as proof for proper orthology assignment. The method asks in principle: if we observe gene order conservation at the level of protein families, do we also observe it at the level of high-resolution orthologous groups. In the specific implementation of the method we define gene families by COGs. We have assigned orthology numbers to all genes in the COGs using LOFT. We refer to these as LOFTyCOGs: high-resolution, multi-level orthology assignments within the COGs. Although a COG often incorporates several genes from a single species, LOFTyCOGs do not. Subsequently we start out by selecting cases where we have gene-order conservation at the level of a (low resolution) COG with a (high resolution) LOFTyCOG, and then examine to which extent the genes from that COG are also from a single LOFTyCOG: i.e. to what extent do LOFTyCOGs correctly form high-resolution sub-clusters from a COG. Accordingly, we require different species to have at least two genes from two corresponding LOFT clusters in the same succession on their genome. The procedure starts by randomly selecting a gene from a COG and testing that against every other species in that COG. In order to avoid simple cases, these species must have at least two genes in the COG. In addition, we require that for every species tested against the selected gene, there exists exactly one gene with strict gene-order conservation at the COG level. This way, it is guaranteed that there is a solution among several candidates. The question is then whether the right assignment is made. The benchmark recognizes several "decreasing" levels of correctness:

Correct: there is full agreement between the orthologs of the successive genes

Member: the ortholog of the succeeding gene is a sub-ortholog (or vice versa), but it is the only candidate with the same level of relatedness.

Ambiguous: the ortholog of the succeeding gene is a sub-ortholog (or vice versa), but there are more candidates at the same level of relatedness.

Related: none of the above, but the ortholog of the succeeding gene is a member of the same base group

Wrong: the ortholog of the succeeding gene is not a member of the same base group

Further details are described in the Methods section.

**Figure 2:**
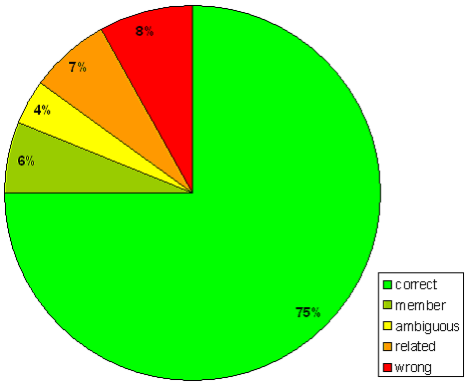
**Benchmark results of LOFT**. Benchmark results of the quality of orthology prediction based on gene order conservation and using LOFT to determine orthology relations (see text for details).

The benchmark is applied to 178 complete genomes, involving a total of 294,011 genes that were assigned to 4,325 different COGs. The results are summarized in Figure 2 which shows that 75% of the cases were classified as 'correct'. In another 6% of the cases, there is only one candidate gene that has a membership relation, which is also the gene with conserved gene-order ('member'). Both categories together, 81%, can be regarded as correct orthology predictions. The benchmark classifies 4% of the cases as 'ambiguous'. These include situations with recent duplications, which always lead to an ambiguous result. Only 8% is classified as 'wrong', while another 7% is only 'related'.

### Single Gene Orthology

The benchmark tests consistency between orthology assignments of two genes, by comparing the assignments of the gene neighborhood for both genes. In order for a case to be classified as correct, both genes must fall in the same orthologous group. This requires both preceding and/or succeeding genes to have equal orthology assignments as well. Accordingly, the percentages presented in the benchmark do not directly concern the respective probabilities for single genes. A fair approximation of the correct orthology probabilities for a single gene can be obtained by taking square roots. This is based on a simplified view on the benchmark requiring that two genes need to be correctly assigned in order to get a correct result. The cumulative fractions for pairs of genes are: 75% correct, 81% member or better, 85% ambiguous or better, and 92% related or better. Taking square roots, these cumulative fractions for single become: 87% correct, 90% correct member or better, 92% ambiguous or better, and 96% related or better. Drawing the line between good and bad between the ambiguous and the related classes, 92% of the individual assignments are estimated to be good, while some 8% appears to be bad.

The benchmark ensures multiple candidates in the COG for the species under consideration (see Methods, *Benchmark procedure*; rule 2). Cases where there is only one candidate are excluded from the benchmark. Yet, even in those cases, this single candidate does not have to be the confirmed ortholog. For these simple cases we found 93% of the orthology predictions to be consistent with gene-order. This is important, as these are the majority of all cases while the benchmark reflects the difficult cases. Again taking the square root, the benchmark indicates that 97% of the individual orthology assignments are correct. On average it seems fair to estimate that 95% of the individual orthology assignments made by our procedure are correct.

### COG Gene-Order Conservation

**Figure 3:**
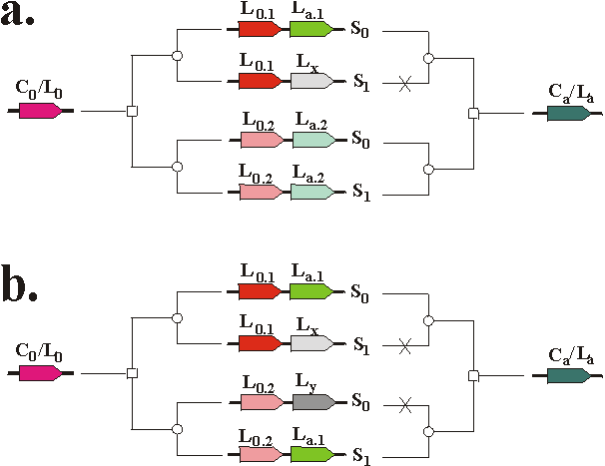
**Misconstruing scenario's**. Misconstruing scenario's: genes C0/L0 (reddish) and Ca/La (greenish) co-duplicate, forming two orthologous groups in two species S0 and S1. a. Gene La.1 is lost in species S1 (gray). On a COG basis, gene L0.2 in species S1 is the only gene with gene-order conservation. Based on LOFT, this scenario is correctly interpreted. b. Gene La.1 is lost in species S1, while gene La.2 is lost in species S0 (differential gene-loss). As a result, gene La.2 in species S1 is erroneously assigned to LOFT cluster La.1 causing gene L0.2 in species S1 to appear the gene with gene order conservation (while using COG or LOFT).

The benchmark uses a strict definition of gene-order conservation: preceding or succeeding genes must not only be in the same COG, but in the same LOFTyCOG, to be accepted as confirmation for orthology by conserved gene order (see Methods, *Benchmark procedure*; rule 3). This requirement can be relaxed by modifying rule 3: rather than requiring that genes in one LOFTyCOG are preceded or followed by a gene from the same LOFTyCOG cluster (L_b _or L_a_), we *only *require them to be preceded/succeeded by a gene from the same COG (C_b _or C_a_). In that case, the benchmark scores actually become considerably *less *good: 45% correct, 8% correct member, 3% ambiguous, 14% related and 30% wrong. We explain this paradoxal result in which relaxing the criteria reduces the quality of the results by two factors. First, by loosening the gene-order requirement (rule 3), many more genes satisfy the inclusion criteria for the benchmark, among which there is a high percentage of genes that possess gene-order conservation on a COG level but not on a LOFTyCOG level. Second is the phenomenon of duplication of gene clusters in combination with gene loss events (Figure 3). Two consecutive genes, that are duplicated together, initially result in the ambiguous situation where two genes show gene-order conservation on a COG basis. Yet, in our benchmark, we exclude situations with more than one gene with conserved gene order. Accordingly, only those situations are kept in which one of the genes is lost.

Consider the scenario of Figure 3a: L_a.1 _is in the same COG as L_a.2_, hence one observes gene-order conservation for genes S_0_/L_0.1 _and S_1_/L_0.2_. However, in our benchmark we require that the LOFT clusters for these genes are equal (they are both either L_0.1 _OR L_0.2_), which they are not. When analyzing the same situation on the basis of LOFT clusters, one correctly finds that S_0_/L_a.1 _does not have an ortholog and the gene-order for S_0_/L_0.1 _is not conserved. Another scenario is presented in Figure 3b. Due to differential gene loss in L_a_, gene S_1_/L_a.1 _obtained the wrong LOFT assignment. The gene with the alleged gene-order conservation is therefore gene S_1_/L_0.2_. Although this scenario in truly problematic, it will be solved by LOFT as soon as another (closely related) species is incorporated in the analysis for which there is no differential gene loss, as this would resolve the unjust assignment of LOFT cluster L_a.1 _to the S_1 _gene and justifiably assign L_a.2_. This is comparable to the *witness gene *concept of Dessimoz et al [28]. The recuperating effect of a third species demonstrates the principal value of tree-based orthology assignment.

### Species Overlap versus Species Tree Reconciliation

Orthology assignment using LOFT critically depends on proper recognition of gene duplication events. For this we have used a simple species overlap rule, allowing full analysis without a priori knowledge of the evolutionary history of the species involved. Yet, species tree reconciliation [18,20-22] is the common method to assess node-type, inferring false duplication events in the case of incongruencies. However, given the continuous debate the issue receives [29], and the high impact of the subject [30], trusted species trees are not always easy to obtain or derive. Furthermore, species tree-reconciliation requires not only for the species tree to be correct, but also the tree of the gene family that is analyzed. Because the species overlap rule is equivalent to species tree reconciliation based on completely unresolved species trees, all duplication nodes discovered by the species overlap rule will also be discovered by species tree reconciliation. On top of that, species tree reconciliation infers additional duplication events in cases where branches have no overlapping species but are incongruent with the topology of the species tree. Vice versa, the species overlap rule may miss gene duplications in cases of complementary gene-loss.

Using the benchmark, we can compare the results obtained by applying species tree reconciliation, with those obtained applying the simple species overlap rule. For this comparison we used a subset of species for which trusted species tree has recently been published: the γ-proteobacteria [31,32]. As some gene-families become very small after the deletion of all genes that do not belong to the γ-proteobacteria, we left out the COGs with fewer than 6 remaining genes, leaving 1006 gene families with a total of 11624 genes.

Species tree reconciliation finds 4554 duplication events in the 1006 trees, 75% (3391) of which are also detected by LOFT's species overlap method. We expect that at least part of the additionally assigned duplications are false and result from noise in the gene-tree reconstruction. Even though species tree reconciliation leads to more duplications, and hence an even higher orthology resolution, the quality of the orthology assignments is not higher than for the species overlap method (Table 1). This indicates that LOFTs species overlap technique is capable of discovering most of the gene duplications that are relevant for orthology assignment.

**Table 1:** Comparing benchmark results for species overlap/species-tree reconciliation

	**Species overlap**	**Species-tree reconciliation**
**correct**	70%	67%
**member**	7%	4%
**ambiguous**	4%	3%
**related**	3%	4%
**wrong**	16%	22%

Benchmark comparison of the species overlap method with the species tree reconciliation method for orthology prediction. The analysis includes 11624 γ-proteobacteriotic genes from 1006 COGs which have at least 6 genes from the γ-proteobacteria. The species overlap method detects 3391 duplications where the species tree reconciliation method detects 4554. Nevertheless, the species overlap method, which does not assume a species tree, has more orthology assignments that can be considered correct (classes "correct" and "member") than the species tree reconciliation method, and can be considered to be at least on par with it.

### LOFT versus COG in the Fungi

In addition to the above, we applied LOFT to 27 complete fungal genomes. In order to do that, we first applied the COG methodology [5,33,34] to obtain gene-families (the FOGs – Fungal Orthologous Groups, results to be described elsewhere). For each of the FOGs we made multiple sequence alignments and derived the phylogenetic tree. The procedure matches that for the COGs as part of the previously described benchmark. Table 2 compares some statistical results of the LOFTyFOGs and the LOFTyCOGs. These statistics show substantial differences. E.g. the number of COGs is about half of the number of FOGs, while it involves many more species and genes. Clearly, and not unexpectedly, the COG methodology is better capable of separating orthologous groups when only analyzing closely related species. As a result of this, the gain in resolution from COGs or FOGs to base groups (the highest order number) is notably smaller in case of the FOGs than in the COGs. Although the statistics differ substantially the percentage of genes covered by unique species/base group combinations is similar (77% for COGs and 82% for FOGs). We found that LOFTyFOGs are much less deeply nested than LOFTyCOGs: where in the COG analysis, 48% of the genes immediately belong to a LOFTyCOG base group (the corresponding branches only have speciations after the last ancient duplication), the FOG analysis places 84% of the genes in a LOFTyFOG base group. The COG analysis leaves 23% of the genes in sub-groups of LOFT level of 4 or higher, while in the FOG analysis this is only 3%. The most straightforward explanation for this is that intermediate duplications are less common within the Fungal clade, as speciation events are relatively recent compared to the species that were incorporated in COG. In our definition, these intermediate duplications give rise to varying levels of orthology. Alternatively, the COG phylogenies could be more error prone due to the wider phylogenetic range of the species included, which may lead to more erroneously assigned gene duplications, and hence, a deeper nesting of orthologous groups.

**Table 2:** Statistics of the LOFT analysis

	**COG**	**FOG**
**Species**	178	27
**Genes**	294011	126230
***genes per species (average)***	*1651.7*	*4675.2*
**Orthologous Groups (trees)**	4325	8740
***genes per OG (average)***	*68.0*	*14.4*
**Base groups**	21130	11082
**Unique spec./base group combinations**	227143	103931
***genes in uniq spec./base group comb*.**	*77%*	*82%*

Statistics of the LOFT analysis of orthologous groups based on COGs and on Fungal Orthologous groups (FOGs). LOFT's parsing of the COGs into high resolution orthologous groups leads to a larger increase in the orthology resolution than the parsing of the FOGs, as reflected in the numbers of base groups. Notably, the FOGs started off with a higher resolution due to the application of the COG methodology on a set of closely related species.

## Discussion

With the introduction of the terms orthologs and paralogs, Fitch expressed the need to describe the nature of gene-relations when dealing with both speciation and gene duplication events [24]. Other terms have been introduced later, like co-ortholog, inparalog and outparalog, as refinements to improve the description of these relations. In more complicated but nevertheless common situations, this terminology is not sufficient to accurately describe the gene-relations that arise from multiple gene duplications and speciations. We have shown here that "*levels of orthology*" concept allows a reliable and high resolution orthology as well as an adequate discretized description of the evolutionary relations between the different orthologous groups. Similar to hierarchical numbering for functional classification, like in E.C. numbers or G.O. clusters, LOFT provides a scalable resolution to orthology. The use of LOFT as a day to day tool is enhanced by its use of colors, where the successive orthologous groups are represented in different colors, and node-types are indicated. This strongly facilitates visual inspection of the trees. Moreover, LOFT is very efficient: complete species tree reconciliation on a large tree (having some 1000 genes) only requires about 2 milliseconds on a modern workstation. LOFT therewith is a practical tool for tree-based orthology prediction, introducing a numbering scheme that allows the efficient exploitation of relative levels of orthology.

The assignment of orthologous groups using LOFT within COG clusters results in high resolution orthology, with, as estimated, 95% proper assignments according to the gene-order conservation benchmark. The aim of the benchmark was to show the usefulness of the concept of levels of orthology: LOFT allowed us to discriminate, in an automated fashion, between the different types of ambiguities one runs into when examining gene order conservation in combination with phylogeny. However, as the benchmark depends on the quality of the alignments and on the phylogenetic methods used, it reflects the accumulated error of all steps involved. Therefore the gene-order based benchmark as measured by LOFT can in principle also be used to compare the quality of the sequence alignment or phylogeny methods using biological sequence data. One might expect other phylogenetic methods, like maximum likelihood or Bayesian techniques to be more accurate than neighbor joining which we used here. "Living in a time of complete genomes" and doing large-scale orthology prediction we do however not always have the possibility to do every aspect of the phylogenetic analysis as thoroughly as one would do it for a single gene family tree. The largest trees in our benchmark (involving 2,437 genes) could e.g. not be analyzed by PhyML [35] nor by MrBayes [36], both among the fastest implementations around for the respective methods, but still quite computationally intensive [37]. In any case, the orthology benchmark shows quite proper results using simple neighbour joining as the phylogenetic tool. One should keep in mind, however, that proper orthology assignment does not require a faultless tree, all that has to be done is to reliably separate the gene duplication events from the speciation events. Because of that, because a species tree is not always available, and because trees of individual gene families will have many errors, irrespective of the method used, we think that for orthology prediction the species-overlap rule is a useful alternative to species tree reconciliation. In any case, the LOFT numbering scheme itself does not depend on the methods used to derive the tree or to assign speciation and duplication events to nodes. It extracts the orthology relations from a given tree allowing the comparisons that we presented here, and, coupled to first-order phylogenetic tools it is a practical tool to obtain reliable orthology at a higher level than what can be obtained by best-hit methods, while describing the relations between orthologous groups.

## Conclusion

The tree-based orthology assignments made by LOFT using standard, large scale phylogenetic methods (Muscle, NJ, gene tree parsimony rooting) appears to be highly reliable. In this paper we estimate that 95% of the individual orthology assignments based on this method are correct. This is remarkably high as it reflects the accumulated noise of all steps involved. For orthology assignment, which requires a course grain analysis of evolutionary history, to correctly separate speciation events from gene duplication events, these standard methods therefore appear of a high enough quality.

The tree-based approach offers a high resolution, while the "*levels of orthology*" concept allows this resolution to be scalable. At the same time, the relations between orthologous groups, as may be clear from a tree, are summarized in the orthology numbers. LOFT assignments are therefore suited for large scale automated phylogenomics.

In order to facilitate a large scale analysis on all 178 species in the COG, we defined a species overlap rule to decide upon speciation versus duplication events in a tree. Based on our gene-order conservation benchmark this species overlap rule performs at least as good as the classical species tree reconciliation. If this could be confirmed on other types of data, species-overlap reconciliation for orthology assignment would become a valid alterative to species-tree reconciliation.

Our gene-order conservation benchmark depends on the quality of all the steps involved, and can therefore be used to compare them. A comparison can in principle be made for alternative tree-generating routines or multiple sequence alignment routines based on real data using this benchmark.

## Methods

### Speciations or Duplications

In a phylogenetic gene tree, we presume all nodes to represent either a duplication event or a speciation event. LOFT can be instructed to do classical species-tree reconciliation as described by Smazek and Eddy [22] and it can be instructed to use its species overlap rule: it considers a node to represent a gene duplication if its branches have overlapping sets of species, i.e. if the branches have genes from a common species. By contrast, if the sets of species of the branches are mutually exclusive, the node is considered to represent a speciation event. After all node-types have been established, the orthologous relations can be deduced: genes are in the same orthologous group when they are separated by speciation events only.

### Ancient, Intermediate, and Recent Duplications

LOFT distinguishes between ancient, recent and intermediate duplications. Ancient duplications took place before the first speciation in the set of homologous genes compared, intermediate duplications took place in between speciations, while recent duplications took place after the last speciation.

### Assigning orthologous group numbers

LOFT starts assigning orthologous group numbers directly after the ancient duplications, before the most ancient speciation in each lineage. Ancient duplications, resulting in ancient paralogs, engender different base numbers for orthologous groups: the "base groups", specifying the base level for orthology within the gene family. Numbering starts at the top with base number 1. Intermediate duplications result in sub-orthologous groups, e.g. 1.1 and 1.2, indicating fully paralogous genes descending from a gene in base group 1. An intermediate duplication of a gene in group 1.2 will result in sub-orthologous groups 1.2.1 and 1.2.2, which are full paralogs descending from a gene in orthologous group 1.2. In the event that *another *gene from orthologous group 1.2 is *also *duplicated, LOFT will assign numbers 1.2.3 and 1.2.4 to these paralogous groups, because numbers 1.2.1 and 1.2.2 have already been assigned.

Although the above procedure is applicable to multifurcating trees, for bifurcating trees it holds that x.y.1 and x.y.2 are direct paralogs, descending from a gene in group x.y, and so are x.y.3 and x.y.4, etcetera. For these bifurcating trees, the LOFT numbers directly distinguish paralogs (e.g. 3.1 and 3.2) from other co-orthologs (like 3.2 and 3.3). In multifurcating trees, the groups 3.1, 3.2 and 3.3 can, but do not have to come from the same (collapsed) duplication event. By definition, recent duplications are not followed by speciation events. Genes that result from a recent duplication are therefore inparalogs, in one species. The LOFT number that is assigned to them will nevertheless indicate their paralogous relation.

### Rooting

As orthology depends on gene evolution and ancestry, the trees that are used to derive orthologous relations require proper rooting. This is often accomplished by including a known outgroup. Using LOFT, trees can be rooted manually by using a popup menu on a node. For automated analyses, one can either use pre-rooted trees, or use the *auto-root *feature of LOFT, which can also be used when no obvious outgroup is present. The *auto-root *feature uses two criteria. First, the program chooses the root that minimizes the number of gene duplications (a type of *gene tree parsimony *[19]): for all possible roots the number of inferred gene duplications is calculated and the root with the minimum number of duplications is selected. If there are multiple solutions with an equal, lowest number of gene duplications, LOFT will select those with a minimal number of intermediate duplications (duplications between speciation events). Finally, the *auto-root *feature applies a midpoint criterion to select the best root among the equally optimal solutions (selects the root for which the difference between the average path to its leaves for both branches is minimal). As every gene duplication gives rise to a new orthologous group, this procedure effectively minimizes the number of orthologous groups derived from the tree. With respect to rooting it is important to understand that root selection does not actually influence the deduced levels of orthology, as long as the true root lies somewhere within the region of ancient duplications, i.e. before the first speciation. Presuming this is the case, root selection may only affect the actually assigned orthologous numbers, not the topology of the gene relations, nor the levels of orthology. Sub-optimal rooting will therefore have little impact on the orthology assignments and the inferred gene-relations [28].

### Gene-order conservation benchmark procedure

**Figure 4:**
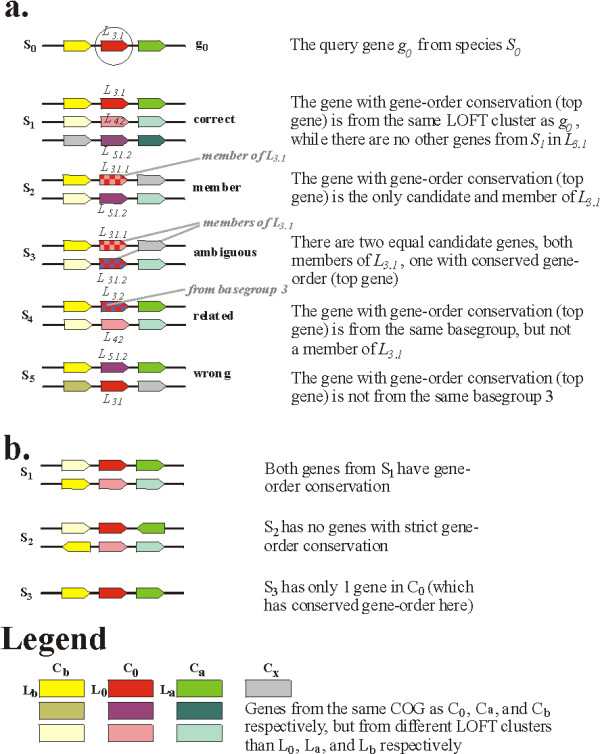
**Benchmark details**. The colors yellow, red and green point to genes from different COGs. Exact color matches indicate genes within the same LOFTyCOG cluster, presumably true orthologs. a. Possible outcomes. Gene g_0 _from species S_0 _is selected for analysis. Its gene neighborhood are genes in LOFT cluster L_b _and L_a_. In all cases, there is exactly one gene with gene order conservation. b. Excluded cases. Species S_1 _is excluded from the benchmark becauseg there are two genes with gene-order conservation; the first conserves L_a_, the second conserves L_b_. Species S_2 _is excluded from the benchmark because there is no strict gene-order conservation due to different directions of transcription. Species S_3 _is excluded from the benchmark because it has only one gene in C_0_; there is no possibility for ambiguity in its selection.

The benchmark procedure (Figure 4) is as follows:

1) Select a random gene (g_0_)

a. Note its species (S_0_), its COG cluster (C_0_), as well as its LOFTyCOG cluster (L_0_).

b. Determine which gene lies after (g_a_- 3'), and before (g_b_- 5') gene g_0 _in species S_0_. Here we consider only prokaryotic genes transcribed in the same direction as g_0 _to ensure conservation over large phylogenetic distances.

c. Note the COG and LOFTyCOG of both neighboring genes (C_a_, C_b_, L_a_, L_b_).

2) Select only those species (S_1_...S_N_) that have at least two genes in C_0_.

3) Make a list of genes (g_x_) from C_0 _that possess gene order conservation; i.e. the gene after g_x _must be from L_a_, and/or the gene before g_x _must be from L_b_.

4) Select only those species that have one and only one gene in C_0 _with conserved gene-order; i.e. that is followed/preceded by a gene in L_a_/L_b _respectively.

The benchmark examines how well L_x _(the LOFTyCOG for gene g_x_) relates to L_0_. The procedure ensures both the possibility of multiple outcomes (rule 2) as well as a single solution (rule 4) in the form of a 'confirmed' ortholog. We consider five possible categories of outcome (Figure 4), representing decreasing levels of correctness, in which we exploit the levels of orthology concept:

*Correct*: L_x _equals L_0 _(the LOFTyCOG from g_0_), the orthology assignment is simply confirmed by a conserved gene order.

*Member*: L_x _is a sub-ortholog from L_0_, but g_x _is the only sub-orthologous gene from the species and therefore the only candidate. This situation occurs when there has been a gene duplication in species S_x _followed by a gene loss. We also allow the reverse situation where L_0 _is a sub-ortholog from L_x_.

*Ambiguous*: L_x _is a sub-ortholog from L_0 _(or vice versa), while the species has more candidate genes which are sub-orthologs at the same level.

*Related*: L_x _is not a sub-ortholog from L_0 _(nor vice versa), but L_x _and L_0 _are from the same orthologous base group (the highest order number, e.g. for 3.1 and 3.6 – see Methods: Assigning orthologous group numbers).

*Wrong*: L_x _and L_0 _do not even belong to the same orthologous base group (e.g. 3.7.2 and 4.1).

For all genes in all COG families [34] we made multiple sequence alignments using Muscle [38]. Next, we generated phylogenetic trees using Neighbor Joining [39] (based on the identity matrix and correcting for multiple substitutions) as a first-order approach. These trees are analyzed with LOFT using its *auto-root *feature, and in absence of a undisputed species-tree for all species in the COGs, the species-overlap rule for deciding upon gene duplication versus speciation events.

The benchmark was carried out on 178 complete genomes, involving a total of 294,011 genes that were assigned to 4,325 different COGs, which we considered gene-families. The largest gene-family, COG0642, held 2,437 genes, while 16 others still have more than 1,000 genes. The multiple sequence alignments, the tree files, the orthology assignments made by LOFT, and a file that lists gene neighbors are all available from the LOFT website[40]. For the benchmark, 3000 genes were randomly selected from different COGs, each compared to as many other species as possible.

## Availability and requirements

Project name: Levels of Orthology From Trees

Project home-page: 

Programming languages: Delphi (Windows version), Java (platform independent)

Licence: GNU

### Note in proof

It has been brought to our attention that a procedure has been published   for iteratively splitting homologous groups into orthologous groups at an   increasing level of resolution, based on a single linkage clustering that   uses correlations between evolutionary distances as a distance measure  [41].

## Authors' contributions

RvdH developed the LOFT concept, programmed LOFT, developed and carried out the benchmark and wrote the majority of the paper. BS contributed to the development of the LOFT concept as well as the benchmark and delivered the gene neighborhood database. VvN delivered the COG clusters as well as the FOG clusters. MH supervised the study, contributed to the paper and critically revised and commented on all aspects of the study. All authors read and approved the final manuscript.
